# Socioeconomic Disparities in Colorectal Cancer Screening in Korea: A Nationwide Cross-Sectional Study

**DOI:** 10.1097/MD.0000000000001368

**Published:** 2015-10-02

**Authors:** Mina Suh, Kui Son Choi, Hoo-Yeon Lee, Myung-Il Hahm, Yoon Young Lee, Jae Kwan Jun, Eun-Cheol Park

**Affiliations:** From the National Cancer Control Institute, National Cancer Center, Goyang, Republic of Korea (MS, KSC, YYL, JKJ); Department of Social Medicine, College of Medicine, Dankook University, Cheonan, Republic of Korea (H-YL); Department of Health Administration and Management, College of Medical Science, Soonchunhyang University, Asan, Republic of Korea (M-IH); and Department of Preventive Medicine, Yonsei University College of Medicine, Seoul, Republic of Korea (E-CP).

## Abstract

Colorectal cancer (CRC) is a common cancer worldwide. The incidence and mortality rates of CRC are higher among lower socioeconomic status (SES) populations.

We investigated the association between different indicators of SES and CRC screening rates in Korea. The eligible study population included males and females aged 50 to 74 years who participated in a nationwide cross-sectional survey (2010–2012). The “compliance with recommendation” category was applicable to participants who had undergone a fecal occult blood test (FOBT), double-contrast barium enema, or colonoscopy within 1, 5, or 10 years, respectively.

In total, 6221 subjects (51.4% female, 55.6% aged 50 years) were included in the final analysis. Lower household income was significantly negatively related to compliance with screening recommendations (*P* for trend < 0.01) and marginally significantly related to noncompliance with recommendations (*P* for trend = 0.07). Older age and poor self-reported health were associated with the screening rate using the FOBT; male sex, older age, higher household income, having supplemental insurance, family history of cancer, and poor self-reported health were associated with a higher screening rate using colonoscopy.

Lower household income was associated with a higher screening rate using the FOBT and with a lower screening rate using colonoscopy. To increase the rate of CRC screening using colonoscopy, efforts should be made toward improving the education and promotion of screening to the low household income target population.

## INTRODUCTION

Colorectal cancer (CRC) is a relatively prevalent cancer worldwide, as it is the third most common cancer in males and the second most common in females.^1^ CRC incidence rates are the highest in Western countries, such as the United States and Canada and across Western Europe,^1,2^ but are either stabilizing or declining in these territories.^3^ Similarly to incidence trends, CRC mortality rates are also decreasing.^4^ This may in part be attributed to improved treatment and increased early detection of CRC.^5^ However, higher CRC incidence and mortality rates among lower socioeconomic status (SES) populations remain.^6,7^

To address this socioeconomic disparity in incidence and mortality rates and to decrease the overall burden associated with CRC, organized screening programs were introduced in several countries during the 2000s.^8^ In contrast to breast or cervical cancer screening, a variety of methods, such as colonoscopy, flexible sigmoidoscopy, double-contrast barium enema (DCBE), and the fecal occult blood test (FOBT), can be used in CRC screening programs. Although colonoscopy represents the most effective method, the majority of organized screening programs for CRC have adopted the FOBT due to limited healthcare resources.^9–12^

In Korea, the incidence and mortality rates of CRC have increased rapidly. In 2010, 25,782 new CRC cases were diagnosed, with an estimated 7645 Korean males and females dying of CRC.^13^ Disparities in the incidence and mortality rates of CRC according to SES are evident in Korea.^14^ In 2004, the National Cancer Screening Program (NCSP) for CRC was initiated. All Korean males and females over 50 years of age are entitled to receive an immunochemical FOBT every year; participants with a positive FOBT result can then undergo further investigation via colonoscopy or DCBE. In addition to the NCSP for CRC, which represents an organized screening program, opportunistic screening using colonoscopy is also available in Korea.^15–17^

Many studies on disparities in cancer screening rates have been conducted worldwide, the majority of which have focused on breast and cervical cancer. In the case of CRC screening, it is possible to choose from various methods, such as colonoscopy, FOBT, and DCBE. Accordingly, the association between SES and CRC screening must be investigated separately according to the screening method. Because repeated screenings at regular intervals are effective, cancer screening recommendations include the most effective intervals. They also need to examine the association between SES and CRC screening by regular follow-up within the interval of the cancer screening recommendation. This study investigated the relationship between different indicators of SES and CRC screening rates in Korea as modified by several factors, including screening modality and regularity, using data from a nationwide survey.

## METHODS

### Subjects

The Korean National Cancer Screening Survey (KNCSS) is a nationwide cross-sectional survey concerned with behavioral patterns associated with cancer screening rates, principally for gastric, liver, colorectal, breast, and cervical cancers. Cancer-free males aged 40 years or over and cancer-free females aged 30 years or over represented the eligible population of the KNCSS. Informed consent was provided by all participants. Details of the survey are described fully elsewhere.^18^ The present study included males and females between 50 and 74 years of age who had participated in the KNCSS between 2010 and 2012. A total of 6221 males and females were included in the final analysis. This study was approved by the Institutional Review Board of the National Cancer Center, Korea (approval no. NCCNCS-08-129).

### Measures

The primary outcome measures of this study were whether participants had ever received FOBT, DCBE, or colonoscopy for CRC screening or if they had undergone a screening test according to the recommendations. Because it is recommended to undergo an FOBT every year, DCBE every 5 years, or colonoscopy every 10 years in Korea, the “compliance with recommendation” category was applicable to participants who had undergone an FOBT, DCBE, or colonoscopy within the last 1, 5, or 10 years, respectively. Table 1 is lacking data for the DCBE category because of an insufficient number of participants (n = 61). Participants who had undergone one or more tests were assigned to categories in the following order of precedence: colonoscopy and FOBT.

**TABLE 1:**
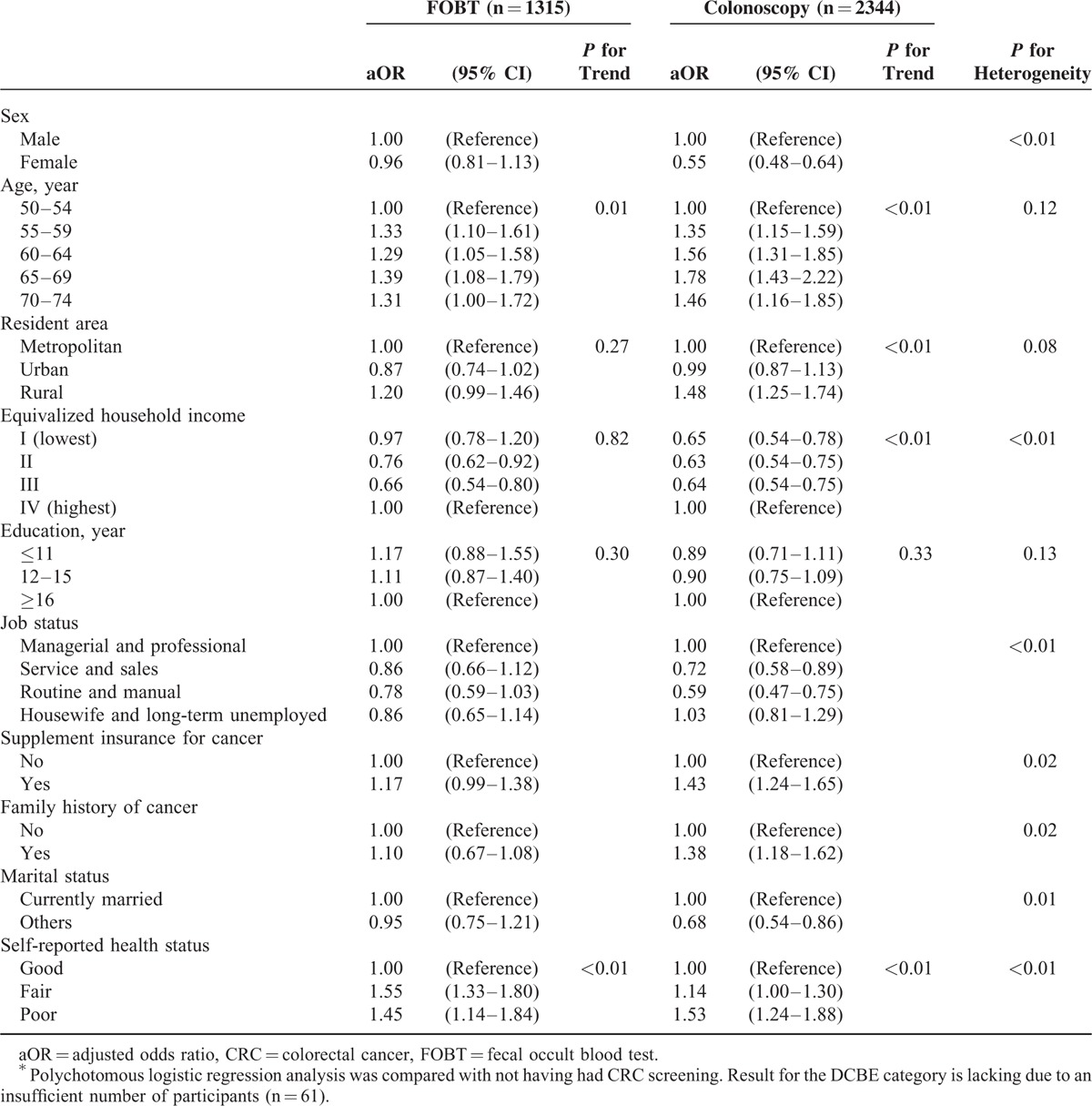
Polychotomous Logistic Regression Analysis of FOBT and Colonoscopy for CRC Screening Compared With Not Having Had CRC Screening^∗^

Independent variables included age, residential area, number of family members, private insurance for cancer, family history of cancer, and marital status. An SES variable, such as the monthly household income, duration of education, self-reported health status, or occupation, was added to the independent variables. For the purposes of this analysis, household income was adjusted according to the number of family members, hereafter referred to as the equivalized household income.^19^

### Statistical Methods

Because the KNCSS was conducted by stratified multistage random sampling, we calculated the weighted proportion adjusted for the sampling rate across the geographic area, age, and sex. The basic characteristics of the participants are presented as unweighted numbers and weighted proportions. Dichotomous logistic regression was used to analyze any associations between indicators of SES and the CRC screening rates. Associations between factors indicative of SES and the regularity and modality of CRC screening were investigated using polychotomous (multinomial) logistic regression. This model allowed for simultaneous odds ratio (OR) estimation for the regularity or modalities of CRC screening and socioeconomic factors with respect to unscreened participants. The Wald statistic was calculated to determine the *P*-value for the heterogeneity of the ORs.^20^ All statistical analyses were performed using the SAS software package (ver. 9.2; SAS, Inc., Cary, NC).

## RESULTS

Table 2 displays the basic characteristics of the 6221 respondents, of whom 51.4% were female and 55.6% were between 50 and 59 years of age. Table 2 also displays the socioeconomic characteristics of the participants: 47.3% had a monthly household income of US $2000 to 3999, 51.5% had 12 to 15 years of education, and 71.4% had private medical insurance for cancer. Good health was self-reported by 57.7% of respondents, with 31.9% reporting that their health was fair and 10.5% reporting that it was poor.

**TABLE 2:**
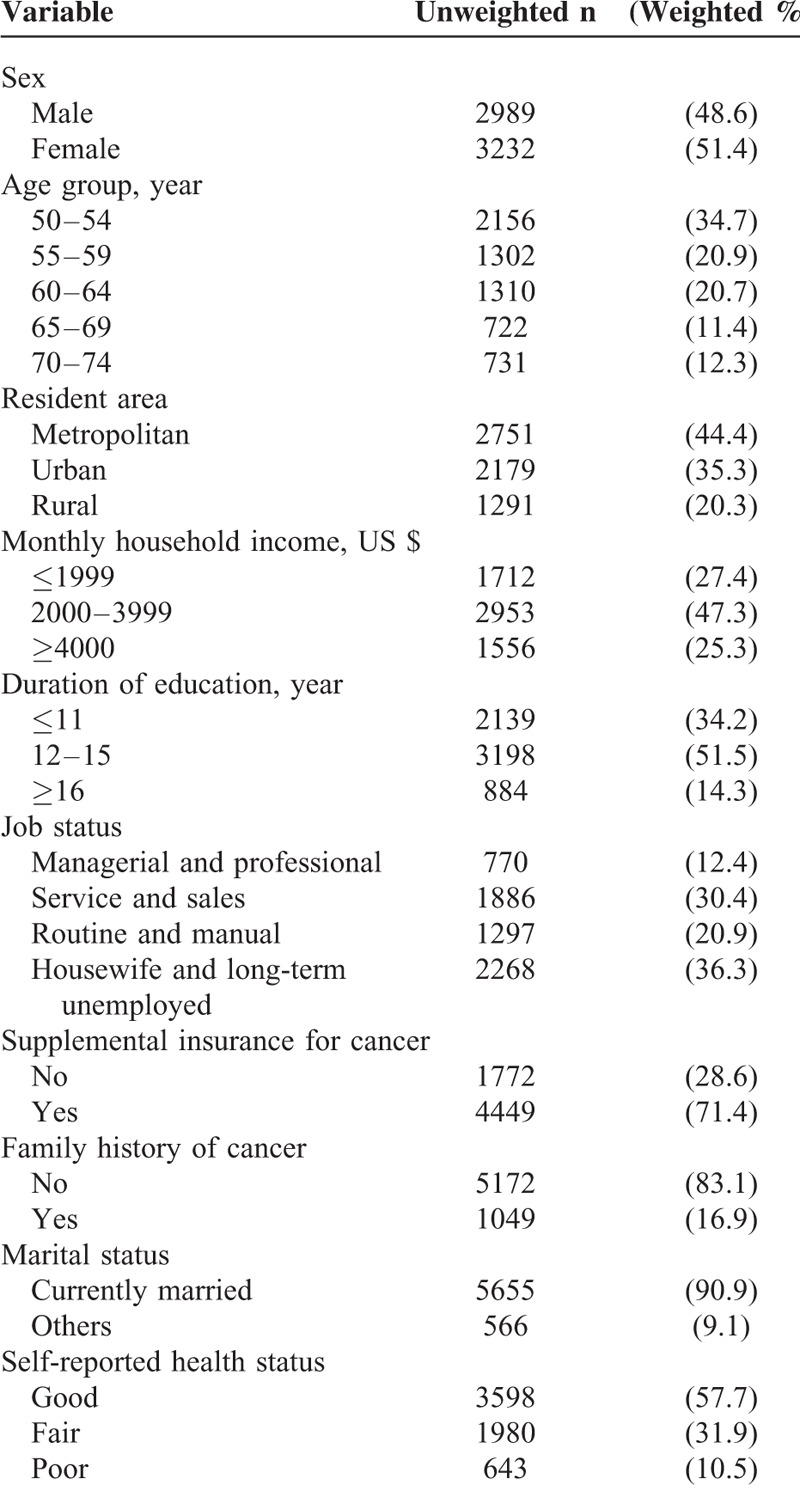
General Characteristics of the Respondents

Male sex and older age were positively associated with having undergone CRC screening in both the crude and adjusted logistic regression analysis (Table 3). Resident area was also associated with having undergone CRC screening. The CRC screening rate in rural areas was higher than in metropolitan or urban areas. Lower equivalized household income exhibited a significant decreasing trend (*P* for trend < 0.01). Education was not associated with having undergone CRC screening. Employment in a service or sales capacity and in routine and manual occupations was associated negatively with having undergone CRC screening, compared with managerial and professional occupations. Supplemental insurance for cancer and family history of cancer were both associated positively with having undergone CRC screening. Poorer self-reported health exhibited a significant increasing trend (*P* for trend < 0.01).

**TABLE 3:**
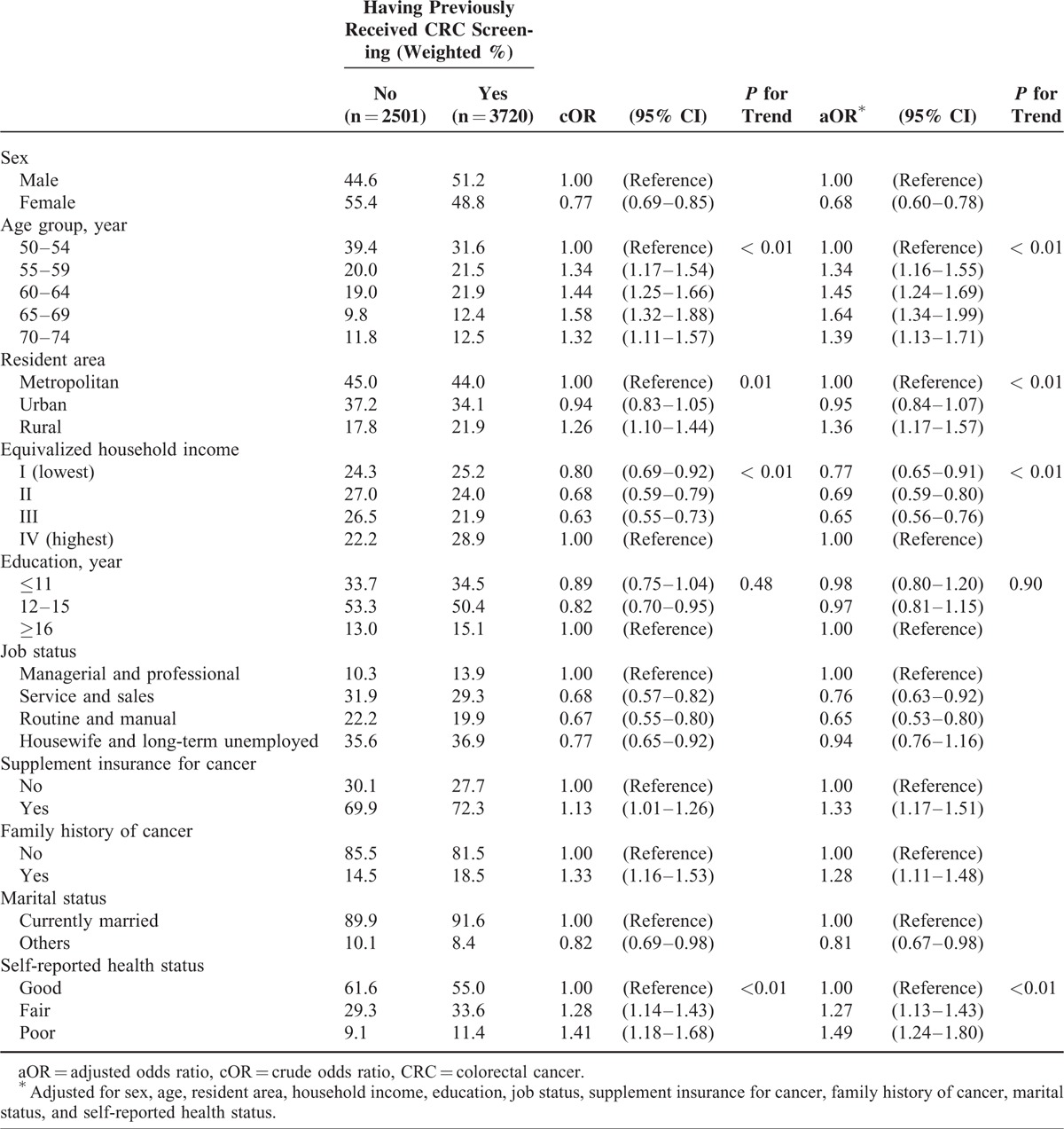
Relationship Between Sociodemographic Characteristics and CRC Screening Status

Table 4 displays the results of the polychotomous logistic regression analysis used to evaluate the socioeconomic factors associated with regularity of and compliance or noncompliance with recommendations for CRC screening. Female sex was negatively associated with both noncompliance and compliance with the recommendations for CRC screening. Older age exhibited a significant increasing trend toward both noncompliance and compliance with screening recommendations. Lower equivalized household income was negatively related to compliance with screening recommendations (*P* for trend < 0.01) and marginally significantly related to noncompliance with screening recommendations (*P* for trend = 0.07). Supplemental insurance for cancer and a family history of cancer status were both associated positively with CRC screening rate, but only in the context of compliance with screening recommendations. Poorer self-reported health was associated with both an increased likelihood of noncompliance and compliance with CRC screening recommendations (*P* for trend < 0.01 for noncompliance; and *P* for trend < 0.01 for compliance).

**TABLE 4:**
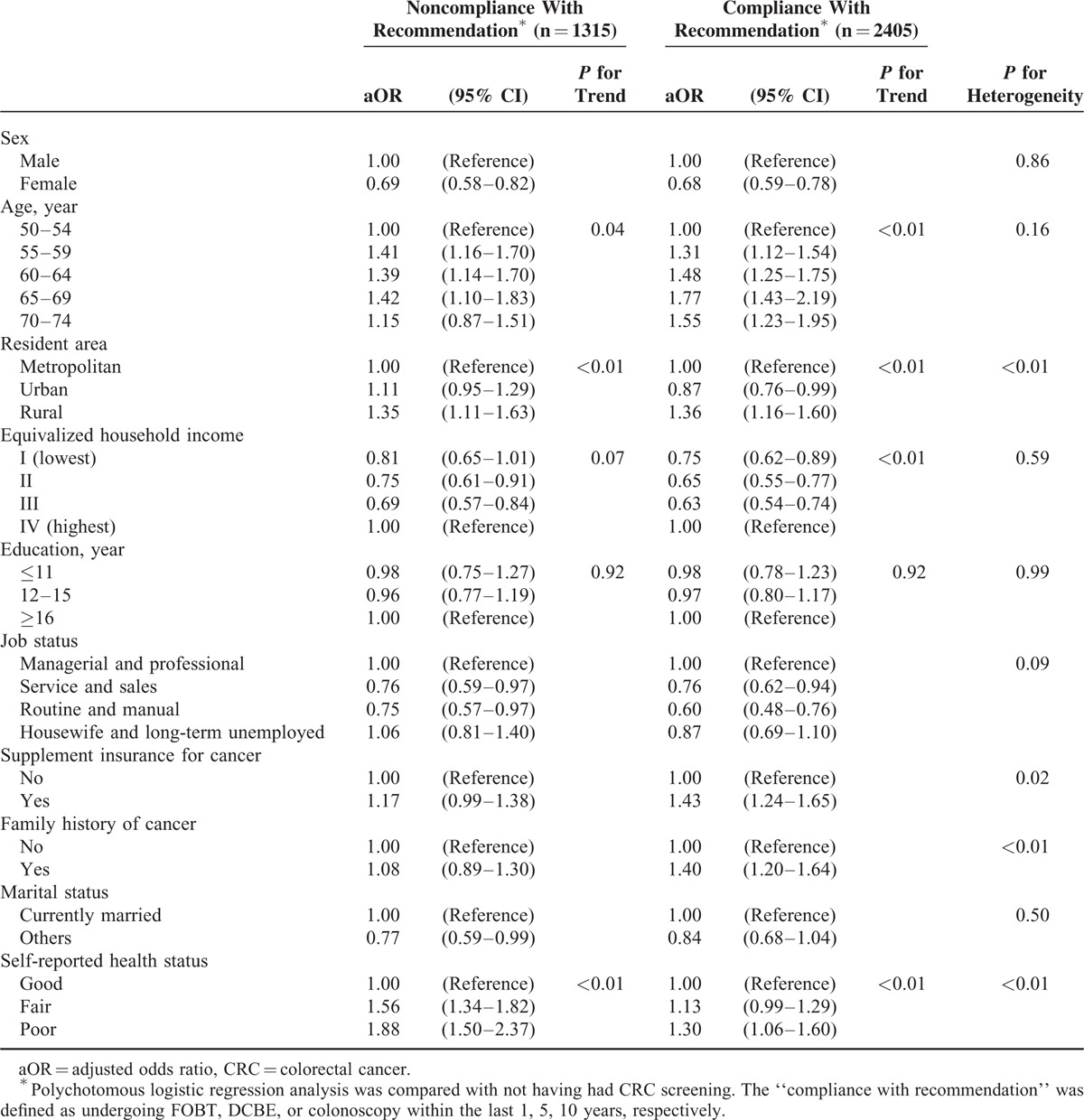
Polychotomous Logistic Regression Analysis of Compliance Versus Noncompliance With Recommendation for CRC Screening

Table 1 presents the results of the polychotomous logistic regression analysis used to evaluate the socioeconomic factors associated with the methods of CRC screening, including the FOBT and colonoscopy, compared with not having undergone CRC screening. Female sex was associated negatively with only colonoscopy for CRC screening. Older age exhibited significant increasing trends for both FOBT and colonoscopy. Residing in a rural area was associated positively with only colonoscopy. Lower equivalized household income exhibited a significant decreasing trend for only colonoscopy for CRC screening (*P* for trend < 0.01). Education status was not associated with either FOBT or colonoscopy. Supplemental insurance for cancer and a family history of cancer status both were associated positively with only colonoscopy for CRC screening. Poorer self-reported health status exhibited significant increasing trends for both FOBT and colonoscopy (*P* for trend < 0.01 for both).

**FIGURE 1:**
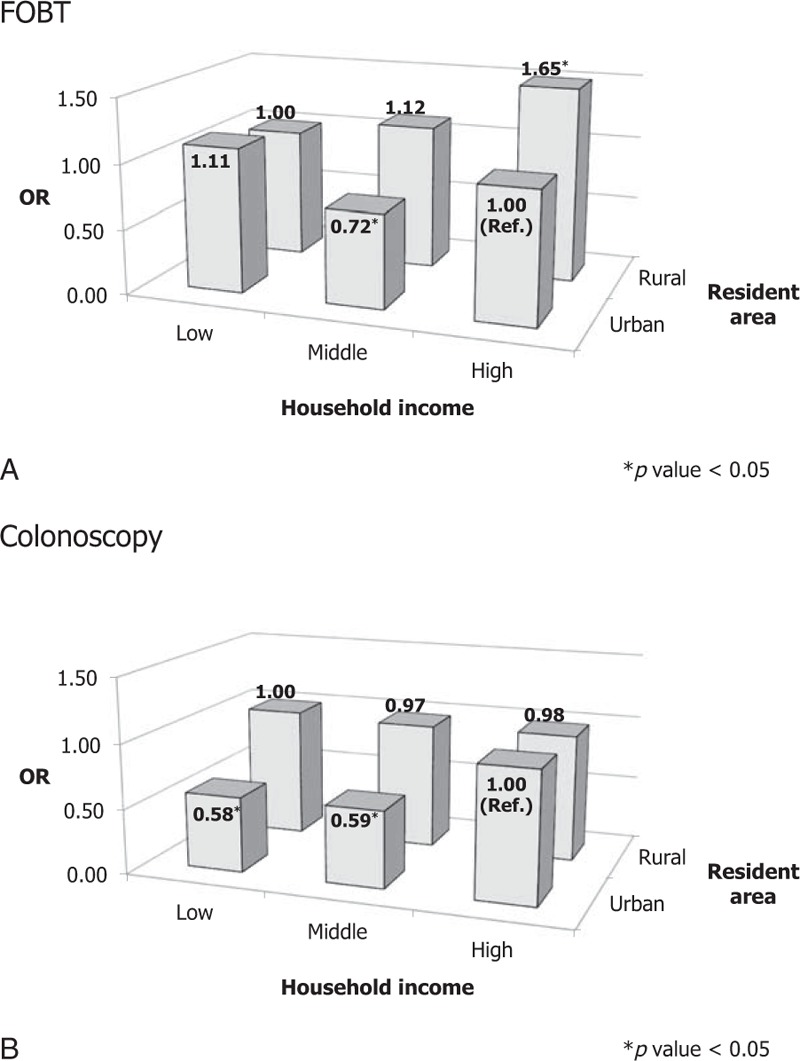
Effect of socioeconomic status on CRC screening using FOBT or colonoscopy.

SES, measured by a combination of residential area and household income, was divided into six categories (Figure 1). There were significant interactions between the residential area and household income variables for both FOBT (*P* < 0.01) and colonoscopy (*P* = 0.02). The CRC screening rate using the FOBT increased continuously with higher household income in those residing in rural areas. In urban areas, the CRC screening rate using colonoscopy decreased commensurate with lower household income. The screening rates using colonoscopy in rural areas did not differ according to household income.

## DISCUSSION

In the present study, male sex, older age, and poor self-reported health were associated with higher CRC screening rates in the context of both compliance and noncompliance with screening recommendations. However, household income, supplemental insurance for cancer, and a family history of cancer were all associated with the CRC screening rate only in the context of compliance with recommendations. In the polychotomous logistic regression model, older age and poor self-reported health were both associated with the screening rate using the FOBT. Male sex, older age, higher household income, supplemental insurance for cancer, a family history of cancer, and poor self-reported health were all associated with a higher screening rate using colonoscopy.

In the KNCSS, CRC screening rates were lower than those reported for gastric, breast, and cervical cancer. Although the lifetime CRC screening rates was reportedly 70.3% in 2013, the lifetime gastric cancer screening rate using gastroscopy or upper gastrointestinal X-rays, lifetime breast cancer screening rate using mammography, and lifetime cervical cancer screening rate using a Pap smear were 80.0%, 83.1%, and 76.2%, respectively.^14^ One previous study suggested that the relatively low screening rate for CRC was due to the complexity of the CRC screening procedure.^21^

In our study, CRC screening rates were higher in males than in females, which is consistent with previous research.^22,23^ Older age was associated with a higher CRC screening rate and exhibited a significant increasing trend toward screening using colonoscopy (*P* for trend < 0.01), but no significant increasing trend toward screening using FOBT (*P* for trend = 0.54). Previous studies are equivocal regarding the impact of age. Although several studies reported similar results,^22,24^ others have suggested the opposite: that younger age is associated with a higher CRC screening rate.^21,25^

Lower CRC screening rates are generally associated with lower SES, as indexed by both education status and income.^23,25–31^ In Korea, the cost of colonoscopy represents a barrier to its use for CRC screening. At slightly over US $60, colonoscopy is >15-fold more expensive than the FOBT test (approximately US $4). In the NCSP, the FOBT test is provided free of charge, whereas colonoscopy is only free for those whose FOBT results are positive. In opportunistic screening, colonoscopy is more expensive than it is in the context of Korean health insurance; moreover, all costs are paid entirely by the individual user, with no governmental subsidy available. Our study demonstrated that possessing supplemental insurance for cancer was also associated with a higher rate of CRC screening.

When equivalized monthly household income and residential area were combined to determine SES, their impact on the CRC screening rate was more marked than the use of either indicator alone. Following the initiation of the NCSP, the disparity in FOBT screening rates, in accordance with SES, is now decreasing.^15^ However, differences still remain according to SES in rural areas. We also observed associations among household income, supplemental insurance for cancer status, and participation rates with the modality of CRC screening used. Although subjects with lower income levels were more likely to have undergone an FOBT, subjects with higher incomes were more likely to have undergone colonoscopy. Therefore, efforts to reduce disparities in screening rates should take into account the area of residence and household income status.

Greater perceived risk of a disease is sometimes associated with positive preventive behavior^32^; in our study, participants with a family history of cancer and poor self-reported health were significantly more likely to have undergone CRC screening. In particular, a family history of cancer was significantly associated with compliance with screening recommendations and with rates of screening using colonoscopy. Anxiety pertaining to being at high risk for CRC (eg, having a family history) and general health anxiety might encourage certain individuals to undergo CRC screening.

Our study has several limitations. First, data collected pertaining to CRC screening participation and SES were self-reported; therefore, although the interviewers received standardized training, recall and interviewer biases remain likely. Because these biases served as a nondifferential misclassification in our study, it would result in bias toward the null. Therefore, our results might be *underestimated*. Second, we employed a cross-sectional design, such that it was not possible to discern causal relationships. Finally, we did not conduct separate analyses according to CRC screening type (eg, NCSPs or private screenings) because of the lack of information regarding the context of the screenings that participants received. Future studies could benefit from recording this information for use in analyses. Despite these limitations, our study is still valuable. We used nationwide data in the context of a stratified, multistage, random sampling procedure in contrast to other studies on CRC screening rate disparities, which have tended to employ smaller samples. Therefore, our results are more representative of the general Korean population.

This study demonstrates that a family history of CRC and self-reported health status are major factors associated with CRC screening rates. Low household income was also associated with lower screening rates using colonoscopy in urban areas. To increase CRC screening rates using colonoscopy, efforts should be made toward improving the education and promotion of screening to the low household income target population. Further research is also required to identify any other remaining barriers to CRC screening in lower-income households.
